# Induced Electron Traps via the PCBM in P(VDF-HFP) Composites to Enhance Dielectric and Energy Storage Performance

**DOI:** 10.3390/polym16213030

**Published:** 2024-10-29

**Authors:** Yantao Yang, Jingqi Qiao, Haiyu Sun, Wenhao Yang, Liangliang Wei, Xuetong Zhao

**Affiliations:** State Key Laboratory of Power Transmission Equipment Technology, School of Electrical Engineering, Chongqing University, Chongqing 400044, China; 202311021135t@stu.cqu.edu.cn (Y.Y.); qjq0411@163.com (J.Q.); yuhaisun21shy@163.com (H.S.); 202311131311@stu.cqu.edu.cn (W.Y.); 202311131158t@stu.cqu.edu.cn (L.W.)

**Keywords:** polymer dielectrics, energy storage, PCBM, electron trap

## Abstract

Polymer-based composites with excellent dielectric properties are essential for advanced energy storage applications. In this work, the [6,6]-phenyl-C_61_-butyric acid methyl ester (PCBM) as a filler was incorporated into the poly(vinylidene fluoride-co-hexafluoropropylene) (P(VDF-HFP)) composite to improve its dielectric performance. P(VDF-HFP) composite films with varying PCBM concentrations were prepared via solution casting and their dielectric, energy storage, and charge–discharge properties were characterized. It was found that the doped PCBM could introduce new charge traps with an energy level of 1.25 eV that modulate charge transport and energy storage characteristics of the polymer matrix. The dielectric constant of the composites was enhanced to the maximum of 10.87 as 0.2 vol% PCBM was added, while the breakdown strength reached 455 MV/m, achieving an energy density of 7.38 J/cm3, which is 33% higher than the pristine P(VDF-HFP) film. Furthermore, the charge–discharge efficiency of the composites was enhanced 66% under the electric field of 300 MV/m. These results demonstrate that PCBM significantly improves the dielectric and energy storage properties of P(VDF-HFP) composites, providing a promising approach for the development of high-performance dielectric materials in flexible energy storage devices.

## 1. Introduction

Dielectric film capacitors are essential components in a range of modern applications, including microelectronics, electric vehicles, and renewable energy generation, owing to their high voltage ratings, flexibility, ease of processing, and environmental benefits [1,2]. Currently, polymer film materials constitute ~60–70% of the total volume of capacitors [3]. Specifically, the biaxially oriented polypropylene (BOPP) becomes the most widely used commercial film material. However, due to its nonpolar molecular structure, BOPP has a relatively low dielectric constant of 2.2–2.3 and a limited energy storage density of 2–3 J/cm^3^, which restricts its potential in advanced energy storage applications [4,5]. For dielectric capacitors, the energy density *U*_d_ of a dielectric material is given by [6]:

(1) $$U_{d}=\int_{D_{r}}^{D_{m}}EdD$$

where *D*_m_ and *D*_r_ are the maximum polarization and remnant polarization, respectively. *E* denotes the applied electrical field and *D* is electric displacement.

For a linear dielectric polymer, *U*_d_ is proportional to the dielectric constant (*ε*_r_) and square of *E*:

(2) $$U_{d}=\frac{1}{2}\varepsilon_{0}\varepsilon_{r}E^{2}$$

where *ε*_0_ is the dielectric constant of vacuum. This equation indicates that energy density is constrained by the dielectric constant and the applied electric field. Thus, research has predominantly focused on increasing the dielectric constant, improving breakdown strength, and optimizing material structure.

To achieve a high *U*_d_, alternative polymer materials with superior dielectric properties have been actively explored. Among them, polyvinylidene fluoride (PVDF) has attracted significant interest due to its high dielectric constant and ferroelectric properties. By copolymerizing PVDF with various monomers, PVDF-based copolymers have been synthesized with improved dielectric performances, making them promising candidates for advanced dielectric applications [7]. Meanwhile, the dielectric and mechanical characteristics of the PVDF-based films can be optimized by adjusting their composition and processing methods to enhance the stability and efficiency under high electric fields [8,9]. Despite these advancements, a single polymer system is increasingly insufficient to meet the growing demands of modern dielectric materials. Researchers have noted that the introduction of functional fillers into polymer matrices is a key strategy to modulate the overall dielectric properties and energy storage capabilities of dielectric composite materials [10,11,12].

Recent research shows a significant stride in improving the performance of polymer-based composites through material design and structural modifications. For instance, Hao et al. [13] incorporated BaTiO_3_ nanoparticles with an average diameter of 6.9 nm into the P(VDF-HFP) matrix to realize high uniform dielectric properties. Similarly, Yuan et al. [14] utilized conductive polyaniline (PANI) as a filler in a PVDF matrix, and an ultrahigh dielectric constant of 385 at 1 kHz was achieved for the composite when the doped PANI content was near the percolation threshold. Additionally, insulating BN nanosheets and semiconductive TiO_2_ were used to inhibit leakage current and space charge conduction in the PVDF-based composites. Zhu et al. [15] demonstrated that an energy barrier was built at the interface between BN and PVDF to prevent electron migration across the BN nanosheets. Wen et al. [16] incorporated single-layer TiO_2_ into PVDF and boosted the energy density of the composite to 21.1 J/cm^3^ under an electric field of 650 MV/m.

However, a mismatch of dielectric constant may occur between nanofillers and the polymer matrix, which may lead to dielectric inhomogeneity and localized field distortion in nanocomposites [9]. Additionally, a high concentration of inorganic fillers often leads to agglomeration and induces an undesired electron conduction at the low electrical field. To address these challenges, a low concentration of organic fillers was introduced to mitigate the common issues of agglomeration and high viscosity in the composites. It is demonstrated that deep traps can be induced with the introduction of organic fillers into the composites, which effectively captures charge carriers, reduces the electric conduction, and thereby enhances the breakdown field [17,18]. Among the organic fillers, the semiconductive [6,6]-phenyl-C_61_-butyric acid methyl ester (PCBM) can capture active electrons because of its high electron affinity [19,20]. It can help to improve the dielectric properties and enhance the energy storage characteristics of the composites.

In this study, poly (vinylidene fluoride-co-hexafluoropropylene) (P(VDF-HFP)) with a moderate dielectric constant (~10) is used as the matrix, and PCBM is introduced as the filler to fabricate the composite. A series of P(VDF-HFP)/PCBM composite films with varying filler concentrations are prepared and the effects of incorporated PCBM on the dielectric properties, energy storage performance, and structural characteristics of the polymer matrix are studied [21,22]. This investigation presents a valuable insight into the interaction between semiconductive organic filler and (P(VDF-HFP)) and provides a feasible route toward optimizing composites for high-performance energy storage applications.

## 2. Materials and Methods

### 2.1. Materials and Film Preparation

The (P(VDF-HFP)) was obtained from Arkema (Colombes, France) and the PCBM was supplied by Macklin (Shanghai, China). N, N-Dimethylformamide (DMF) was purchased from Sigma-Aldrich (St. Louis, MO, USA). Anhydrous ethanol was provided from Chengdu Kelong Chemical Reagent Plant (Chengdu, China).

**Figure 1:**
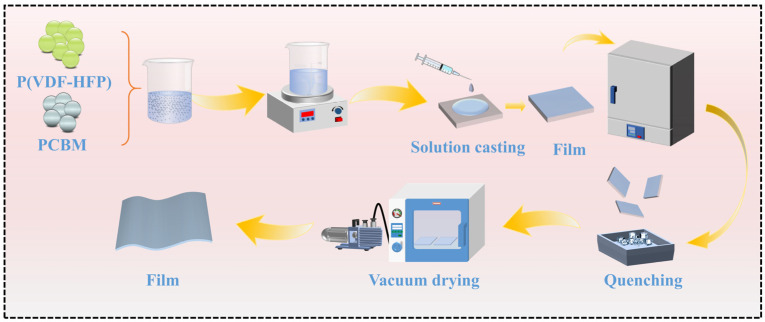
Schematic of the P(VDF-HFP)/PCBM composite films.

As schematically shown in Figure 1, the P(VDF-HFP)/PCBM composite films were prepared using a solution casting method. First, 350 mg of P(VDF-HFP) powder was dissolved in 5 mL of DMF at room temperature with magnetic stirring for more than 6 h until a clear solution was obtained. Meanwhile, 0.77 mg of PCBM was dispersed in DMF through slow stirring for 4 h. The PCBM solution was then mixed with the P(VDF-HFP) solution at varying volume ratios, followed by thorough stirring to ensure homogeneous dispersion. The resultant mixtures were labeled according to the PCBM content, denoted as P(VDF-HFP)/*x* vol% PCBM (*x* = 0, 0.05, 0.1, 0.2, and 0.4). Subsequently, the mixed solutions were cast onto clean glass substrates and dried at 70 °C for 12 h to evaporate the solvent. Afterwards, the films were heated to 200 °C for 10 min then rapidly quenched in ice water. The films were subsequently separated from the glass plates and placed in a vacuum oven at 105 °C for 30 h to complete the drying process. The final composite films show a uniform thickness of approximately 11 ± 2 μm, as measured using the Millimar C1200 thickness gauge (Mahr, Goettingen, Germany).

### 2.2. Characterization

The cross-sectional morphology of the composite film was captured at 2000× magnification with a Quattro S (Thermo Fisher Scientific, Waltham, MA, USA) field emission SEM. X-ray diffraction (XRD) analysis was performed using a Rigaku D/Max 2500PC (Rigaku Corporation, Tokyo, Japan) X-ray diffractometer with a scanning range of 10° to 80° and a scanning speed of 5°/min. Post-processing for peak identification and deconvolution was carried out using Jade 9 software.

Fourier-transform infrared (FTIR) spectroscopy was measured using a Nicolet iS5 spectrometer (Thermo Fisher Scientific, Waltham, MA, USA) in attenuated total reflectance (ATR) mode, with a wavenumber range of 400–4000 cm^−1^. Thermal properties were analyzed via differential scanning calorimetry (DSC) using a Mettler DSC-3 system (Mettler-Toledo, Columbus, GA, USA). Film samples (4–6 mg) were placed in aluminum crucibles and heated from 25 °C to 250 °C at a rate of 10 °C/min under a nitrogen atmosphere to obtain the melting curves, followed by cooling to 25 °C at the same rate to obtain crystallization curves.

Thin Au films of ~30 nm were sputtered as electrodes by a sputter coater (Quorum Q150T (Quorum Technologies, Laughton, UK)) for electrical measurements. Dielectric properties were measured using a Novocontrol Concept 80 (Novocontrol Technologies, Montabaur, Germany) broadband dielectric spectrometer under an applied AC voltage of 1 V, with a frequency range of 1~10^6^ Hz. The ferroelectric test system (PolyK CPE2020-AI-20kV (PolyK Technologies, PA, USA)) was employed to evaluate the electric displacement–electric field (*D*-*E*) hysteresis loops of the composite films. Samples were immersed in Galden HT insulating oil during the measurement to prevent air breakdown.

Leakage current measurements were performed using a Keithley 6517B electrometer (Keithley Instruments, Solon, OH, USA), with voltage and temperature controlled by the Trek 610E voltage (Advanced Energy, Lockport, NY, USA) source and a Delta oven (Keithley Instruments, Solon, OH, USA). Thermally stimulated depolarization current (TSDC) measurements were conducted to analyze the internal charge traps within the dielectric films, utilizing an HC650T (Huace Testing Instrument, Suzhou, China) temperature-controlled stage and a Keithley 6517B electrometer.

## 3. Results and Discussion

### 3.1. Physicochemical Property

**Figure 2:**
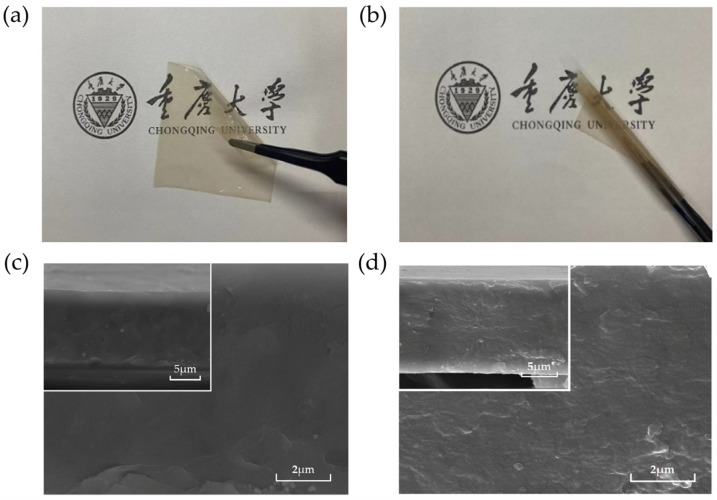
(**a**,**b**) Surface photograph of P(VDF-HFP)/PCBM composite films; SEM images of the cross-sections: (**c**) P(VDF-HFP) and (**d**) P(VDF-HFP)/PCBM composite films.

The surface photograph of the P(VDF-HFP)/PCBM composite films is depicted in Figure 2a,b. Uniform thickness, a smooth surface, and the absence of defects are desired for high-quality dielectric films, and the prepared films exhibit these characteristics, with a thickness of approximately 11 ± 2 μm. As shown in the SEM images in Figure 2c,d, the cross-sectional structure of the composite films is smooth, while the inclusion of PCBM does not introduce any visible pores or defects, indicating excellent compatibility between the organic filler and the polymer matrix. This uniform dispersion of PCBM throughout the film avoids the common agglomeration issues resulting from traditional inorganic nanofillers. The excellent compatibility can be attributed to the good solubility and short molecular chains of PCBM, which effectively fill the micropores between polymer chains, thereby reducing the structural defects [23].

**Figure 3:**
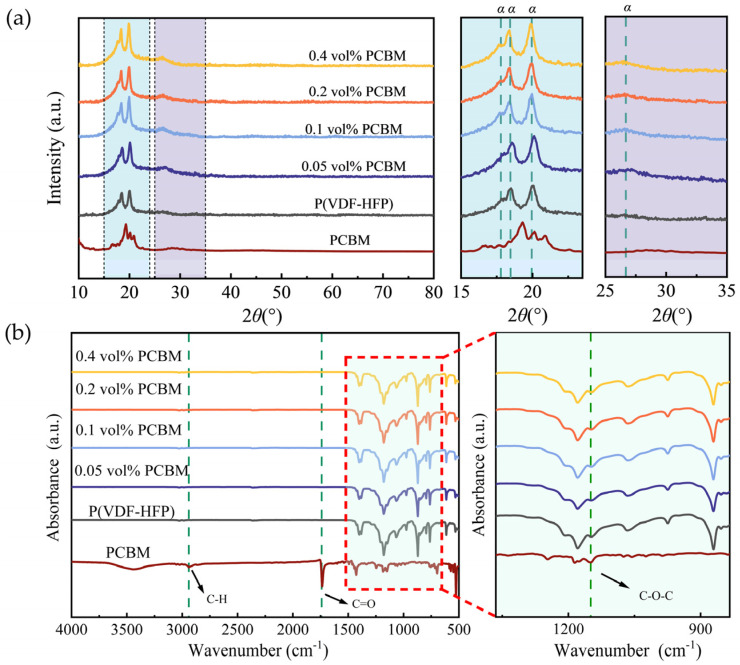
(**a**) XRD patterns and (**b**) FT-IR spectra of P(VDF-HFP), PCBM, and P(VDF-HFP)/PCBM composite films.

As displayed in Figure 3a, P(VDF-HFP) is a semicrystalline copolymer with characteristic diffraction peaks at 2*θ* = 17.7°, 18.4°, 19.9°, and 26.6°, corresponding to the (100), (020), (110), and (021) planes of the monoclinic α-phase of PVDF, respectively [24]. The crystalline regions in the P(VDF-HFP) matrix are predominant in the α-phase. For the P(VDF-HFP)/PCBM composites, the XRD patterns exhibit no obvious changes in the crystalline phases, indicating that low concentrations of PCBM do not alter the crystal structure of P(VDF-HFP).

The Fourier-transform infrared (FTIR) analysis was employed to further validate the crystalline phases and molecular chain conformations obtained from X-ray diffraction (XRD). In the FTIR spectra, characteristic absorption peaks arise from oscillations of the polymer backbone and attached functional groups. As shown in Figure 3b, prominent peaks were observed at 530 cm^−1^, 615 cm^−1^, 763 cm^−1^, 795 cm^−1^, and 976 cm^−1^, corresponding to the asymmetric stretching vibrations of the nonpolar α-phase in P(VDF-HFP) [25,26,27]. These results also confirm that the α-phase dominates the crystalline structure in both pristine P(VDF-HFP) and its composites. For PCBM, characteristic peaks at 2940 cm^−1^ (C–H stretching), 1740 cm^−1^ (C=O stretching), and 1144 cm^−1^ (C–O–C stretching) were expected based on previous studies [23]. However, these peaks were not distinctly observed in the FTIR spectra of the P(VDF-HFP)/PCBM composites due to the low concentration of PCBM or overlap between PCBM and P(VDF-HFP) absorption bands. This suggests that the incorporation of small amounts of PCBM does not significantly change the chemical structure of the composite films [28].

The thermal properties of the materials were analyzed by differential scanning calorimeter (DSC) (see Supporting Materials S1). Briefly, DSC results show that PCBM can accelerate the nucleation process [29,30], resulting in an increase in crystallinity, which is beneficial to improving the mechanical strength and breakdown strength of the composites.

### 3.2. Dielectric Properties

**Figure 4:**
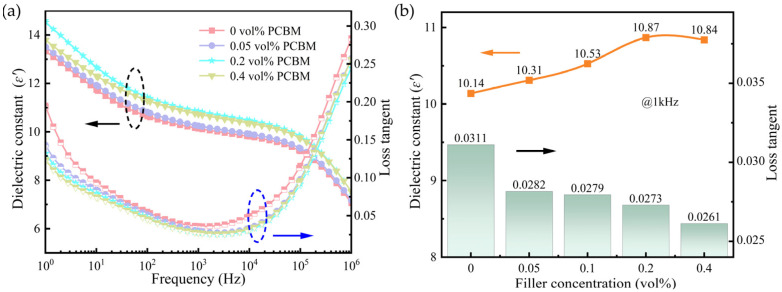
Dielectric properties of P(VDF-HFP)/PCBM composites: (**a**) frequency dependence of dielectric constant and loss tangent; (**b**) dielectric constant and loss tangent at 1 kHz.

Figure 4a illustrates the dielectric properties of P(VDF-HFP)/PCBM composite films at room temperature. The variations in the dielectric constant (*ε’*) and loss tangent (tan *δ*) are plotted as a function of frequency from 1 Hz to 10^6^ Hz. It is evident that the *ε’* of the composite exhibits a strong frequency dependence across the measured frequency range. At lower frequencies, the accumulation of charges at the electrode–composite interface, along with space charge effects, significantly enhances the polarization and produces a high *ε’*. As the frequency increases, the *ε’* gradually decreases. This decline can be primarily attributed to the inability of dipoles to reorient in response to the rapidly changing electric field, leading to dielectric relaxation and a consequent sharp reduction in *ε’* [31,32].

Figure 4b summarizes the *ε’* and tan *δ* at 1 kHz for the composite films with different PCBM volume fractions. The results demonstrate that the dielectric constant of P(VDF-HFP)/PCBM composites increases initially and then decreases as the doped PCBM content increases. Specifically, at a PCBM concentration of 0.2 vol%, the *ε’* reaches the peak value of 10.87, which is approximately 7.2% higher than the value of 10.14 for pristine P(VDF-HFP) films. This enhancement can be attributed to the introduction of the organic semiconductor PCBM, which not only introduces interfacial polarization but also contributes to electronic displacement polarization, thereby improving the overall *ε’* [23,33].

Dielectric loss is typically classified into two categories: polarization loss and conduction loss. In P(VDF-HFP)-based ferroelectric materials, energy dissipation during the polarization and depolarization process is predominantly caused by the volume effect of ferroelectric domains and their interactions, which is commonly referred to as polarization loss [34]. Additionally, free charge carriers within the polymer matrix experience collisions and trapping during their migration and will contribute to the conduction loss. At lower frequencies, the dielectric loss is predominantly governed by the conduction loss. However, as the frequency increases, conduction loss diminishes, while the loss resulting from dipolar orientation polarization and interfacial polarization will play a leading role [35,36,37]. Notably, the loss tangent of the composite films shows a consistent decrease with increasing PCBM content at 1 kHz. For the sample with 0.4 vol% PCBM, the dielectric loss decreases to 0.0261. The high electron affinity of PCBM allows it to effectively trap charge carriers and reduce charge mobility within the polymer matrix. As a result, compared to the pristine P(VDF-HFP) matrix, the incorporation of PCBM significantly mitigates conduction loss and improves the dielectric performance of the composite films.

### 3.3. Energy Storage Properties

**Figure 5:**
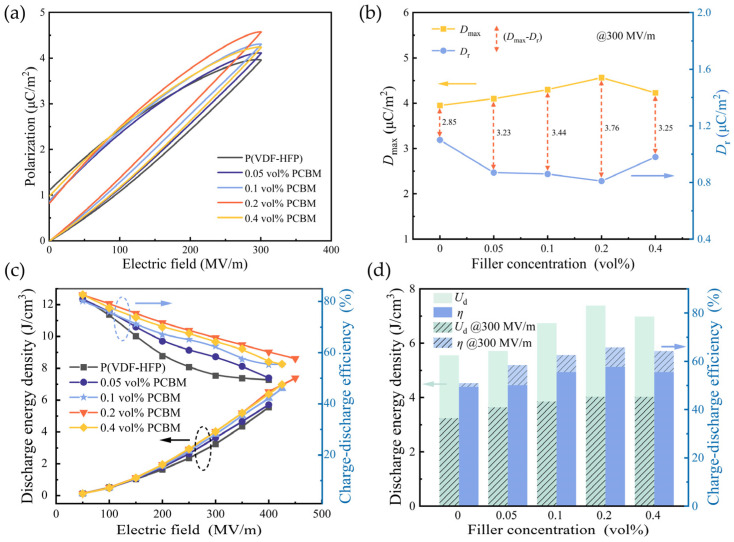
(**a**) The unipolar *D*-*E* loops, (**b**) the maximum electrical displacement and remnant polarization at 300 MV/m electric field, (**c**) the discharge energy density and charge–discharge efficiency, and (**d**) the maximum discharge energy density and charge–discharge efficiency at 300 MV/m for P(VDF-HFP)/PCBM composites.

The electric displacement–electric field (*D*-*E*) loops of the composite films were examined at room temperature. As shown in Figure 5a, compared to the pristine P(VDF-HFP), the *D*-*E* loop of the P(VDF-HFP)/PCBM composite films remains relatively unchanged in shape. For the pure P(VDF-HFP), maximum electric displacement (*D*_max_) is 3.95 μC/cm^2^, a value determined by the nonpolar α-phase characteristics. As the PCBM content increases, *D*_max_ firstly rises and reaches a peak of 4.56 μC/cm^2^ at 0.2 vol% due to enhanced interfacial polarization. However, when the PCBM concentration goes up to 0.4 vol%, *D*_max_ starts to decrease, reflecting a similar scenario of the dielectric constant. It indicates that high PCBM particles may aggregate in the matrix, which may lead to a decrease in the effective interfacial area and weaken the interfacial polarization effect [38]. Additionally, a high PCBM concentration may introduce defects that reduce overall polarization and distort the electrical distribution under an external electric field in the matrix [39,40].

Figure 5b illustrates the variation in *D*_max_ and remnant displacement (*D*_r_) with PCBM filler contents at an applied electric field of 300 MV/m. The difference (*D*_max_ − *D*_r_) between *D*_max_ and *D*_r_ serves as a crucial indicator of energy storage density. As the PCBM content increases, *D*_max_ − *D*_r_ initially rises and then declines. At a PCBM content of 0.2 vol%, the *D*_max_ − *D*_r_ value reaches a maximum of 3.75 μC/cm^2^, indicating that the composite film achieves an optimal energy storage density (*U*_d_) at this concentration.

The energy density *U*_d_ of the composite film is estimated from the unipolar *D*–*E* loop. In Figure 5c, the *U*_d_ of both the P(VDF-HFP) and the P(VDF-HFP)/PCBM composites increases with the external electric field. However, the variation in *U*_d_ exhibits a nonlinear trend with increasing PCBM content, initially rising and then decreasing. The pristine P(VDF-HFP) sample shows a *U*_d_ of only 5.55 J/cm^3^ under the electric field of 400 MV/m. In contrast, the P(VDF-HFP)/0.2 vol% PCBM composite exhibits the highest *U*_d_ of 7.38 J/cm^3^ at 450 MV/m, 33% higher than the undoped sample. This enhancement can be attributed to the combined effects of an increased dielectric constant and improved breakdown strength. However, the *U*_d_ of P(VDF-HFP)/0.4 vol% PCBM declines to 7.13 J/cm^3^ as more PCBM was doped. It is believed that an excess of PCBM doping may disrupt its homogeneous distribution in the polymer matrix. As the PCBM content continues to increase, the breakdown strength gradually decreases. This reduction in performance is attributed to the higher doping levels, which reduce the average spacing between adjacent molecular semiconductors, thereby increasing the likelihood of electron conduction by excited charge carriers. Consequently, the breakdown performance of the composite films is deteriorated [23]. The adverse effects counteract the benefits from enhanced polarization by doping PCBM, ultimately resulting in the decline in *U*_d_.

The charge–discharge efficiency (*η*) is calculated by *η* = *U*_d_/*U*, where *U* represents the energy density that includes both energy loss and discharge energy density [41]. Figure 5d presents the optimal *U*_d_ and *η* at 300 MV/m for the composites with different PCBM dopants. Apparently, the addition of PCBM leads to a marked improvement in *η* across all composite films compared to the pure P(VDF-HFP). At 300 MV/m, the *η* of pure P(VDF-HFP) is only 51%, whereas the composite containing 0.2 vol% PCBM retains a high efficiency of 66%.

As the applied electric field increases, the *η* of the P(VDF-HFP) films tends to decrease due to the intensified charge carrier injection and migration under the high electric field. Furthermore, the increase in residual polarization and energy dissipation during dipoles orientation further lowers the *η* [42]. In contrast, the ability of PCBM to trap charges helps suppress conduction currents, thereby mitigating the conductive loss. This reduction in energy dissipation during charge–discharge cycles allows the composite films to maintain a higher efficiency under high electric fields.

### 3.4. Electrical Breakdown Properties

**Figure 6:**
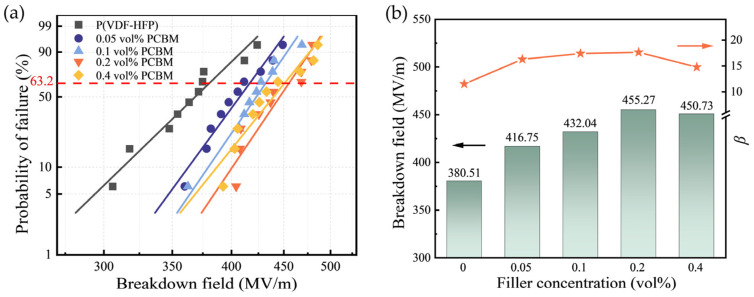
Breakdown field comparison for P(VDF-HFP)/PCBM composites, (**a**) Weibull distribution of breakdown strength, and (**b**) breakdown field and shape parameter *β*.

The electrical breakdown is analyzed using a two-parameter Weibull distribution as shown in Equation (3) [43].

(3) $$P(E)=1-\exp\left(-{\left(E/E_{b}\right)}^{\beta }\right)$$

where *P* (*E*) refers to the cumulative breakdown probability and *E* denotes the measured breakdown strength. The characteristic breakdown strength *E*_b_ is defined as the electric field value at which the probability of electrical failure reaches 63.2%. Additionally, *β* denotes the slope of the Weibull fitting, representing the dispersion of the experimental breakdown strength. As depicted in Figure 6a,b, the *E*_b_ is enhanced from 380.51 MV/m of the P(VDF-HFP) to 455.27 MV/m of the P(VDF-HFP)/0.2 vol% PCBM composite, a 19.65% enhancement in the breakdown strength. It is worth mentioning that the *β* values of composite films are all greater than 10, indicating that the data dispersion is small.

**Figure 7:**
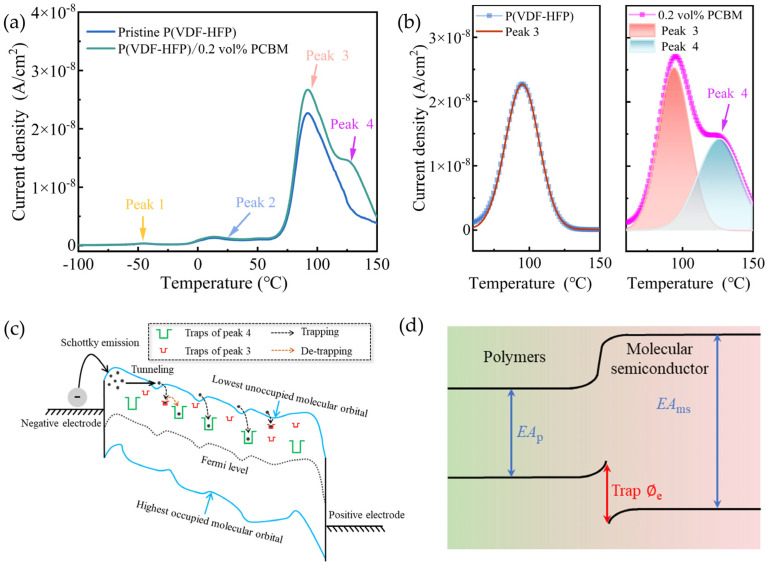
(**a**) Thermally stimulated current spectra of P(VDF-HFP) and P(VDF-HFP)/0.2 vol% PCBM. (**b**) Gaussian fitting of thermally stimulated current peak 3 and peak 4 in the P(VDF-HFP) and P(VDF-HFP)/0.2 vol% PCBM. (**c**) Schematic illustration of trap energy level introduced by the molecular semiconductor of PCBM. (**d**) Trap energy level introduced by the molecular semiconductors. The trap energy level can be calculated using *Φ*_e_ = *EA*_ms_ − *EA*_p_, where *EA*_ms_ and *EA*_p_ are the electron affinities of the molecular semiconductor and the dielectric polymer, respectively.

As shown in Figure 7a, the thermally stimulated depolarization current (TSDC) spectrum of P(VDF-HFP) films reveals three characteristic peaks. Peak 1, occurring around −50 °C, is associated with the depolarization of the polarized amorphous P(VDF-HFP) via devitrification at the glass transition temperature (*T*_g_) [44]. Peak 2, located in the range of 20~50 °C, is related to the depolarization of charges injected from electrodes because of Schottky or thermionic emission [44]. Peak 3, which was found near 100 °C in PVDF, corresponds to the depolarization of trapped charges at the amorphous–crystalline interface [45].

In addition to the three peaks, the TSDC spectrum of the P(VDF-HFP)/PCBM composite shows a new peak 4 around 125 °C, which is absent in the pure P(VDF-HFP) samples. This unique peak 4 indicates the presence of additional charge traps introduced by the PCBM. Figure 7b shows the Gaussian fitting of thermally stimulated current spectra regarding P(VDF-HFP) and P(VDF-HFP)/0.2vol% PCBM.

In general, the current released during the thermally stimulated depolarization process can be described by the following equation [46]:

(4) $$I\left(T\right)=P_{0}\frac{1}{\tau_{0}}\exp(-\frac{E_{a}}{kT})\exp[-\frac{1}{\beta \tau_{0}}\int_{T_{0}}^{T}\exp(-\frac{E_{a}}{kT})dT].$$

where *I* represents the thermally stimulated current, *P*_0_ is the equilibrium polarization strength, *τ*_0_ is the characteristic relaxation time, *k* is the Boltzmann constant (1.38 × 10^−23^ J/K), *T*_0_ is the initial temperature, *T* is the absolute temperature, *E*_a_ is the activation energy of the traps, and *β* is the ramp rate of temperature. Using this equation, the trap energy level associated with peak 3 is ~1.14 eV, while the trap energy level associated with the new peak 4 is ~1.25 eV. It is evident that the introduction of PCBM results in deeper trap levels. It is further demonstrated that the incorporation of PCBM induces new traps that effectively inhibit charge carrier transport and, thus, reduce the conduction loss and enhance the breakdown strength of the composite.

Figure 7c illustrates the traps associated with TSDC peaks of the P(VDF-HFP)/PCBM composite. Specifically, the red region represents intrinsic traps formed at the crystalline/amorphous interfaces of P(VDF-HFP), while the green region corresponds to the new traps introduced by the addition of PCBM. By introducing PCBM into the P(VDF-HFP) matrix, trap energy is established due to their energy band difference, which enhances breakdown strength. The molecular semiconductor of PCBM possesses high electron affinities (*EA*_ms_) compared to those of the dielectric polymers (*EA*_p_), which enables PCBM to effectively capture injected and excited electrons through strong electrostatic attraction, creating deep traps in the composite. The resulting large trap energy level (*Φ*_e_ = *EA*_ms_ − *EA*_p_) restricts the movement of charge carriers, inhibiting the conduction process, as illustrated in Figure 7d [28,47,48].

However, as the PCBM content increases, the semiconducting nature of PCBM begins to exert a greater influence on the breakdown strength. With the filler loading higher than 0.4 vol%, the average distance between PCBM particles decreases, allowing charges to hop more easily under high electric fields [49]. As a result, once the PCBM concentration exceeds a certain threshold, the insulating performance of the composite deteriorates, resulting in a decrease in breakdown strength [50]. As shown in Figure 6b, when the doping content of PCBM is increased to 0.4 vol%, *E*_b_ decreases from 455.27 MV/m to 450.73 MV/m, though it remains higher than that of the pristine P(VDF-HFP).

Furthermore, the leakage current under an applied electric field of 50 MV/m is shown in Figure S2a,b (Supplementary Materials S2). The leakage current density of the pristine P(VDF-HFP) is 8.92 × 10^−8^ A/cm^2^, while the leakage current density of the composite with 0.2 vol% PCBM is significantly lowered to 1.49 × 10^−8^ A/cm^2^. However, as the PCBM content increases further, the leakage current density begins to rise. At a doping level of 0.4 vol% PCBM, the leakage current density reaches 1.59 × 10^−8^ A/cm^2^, which may weaken the trapping effect of PCBM and reduce its ability to capture charge carriers.

## 4. Conclusions

In conclusion, we have successfully developed a series of P(VDF-HFP)/PCBM composite films using a solution-casting method. The inclusion of PCBM as a filler significantly improved the dielectric and energy storage properties of the composite materials. Notably, 0.2 vol% concentration of PCBM in the P(VDF-HFP) matrix produces the highest energy storage density of 7.38 J/cm^3^, which is 33% higher than that of the pristine P(VDF-HFP). This enhancement can be attributed to the synergistic effects of increased dielectric constant and improved breakdown strength, driven by the interfacial polarization and deep traps introduced by PCBM. Furthermore, the optimized composite films demonstrated an enhanced charge–discharge efficiency, reaching up to 66% at an electric field of 300 MV/m. This work provides a promising pathway for the development of high-performance dielectric materials with improved energy storage capabilities.

## Data Availability

The original contributions presented in the study are included in the article/Supplementary Material, further inquiries can be directed to the corresponding author.

## Supplementary Materials

The following supporting information can be downloaded at: https://www.mdpi.com/article/10.3390/polym16213030/s1, Figure S1: DSC curve of P(VDF-HFP)/PCBM composites: (a) melting curve; (b) crystallization curve; Figure S2: (a) Thermally stimulated current spectra of P(VDF-HFP) and P(VDF-HFP)/0.2 vol% PCBM. (b) Gaussian fitting of thermally stimulated current spectra about P(VDF-HFP) and P(VDF-HFP)/0.2 vol% PCBM; Table S1: Calculated DSC curves of P(VDF-HFP)/PCBM composites. References [51,52,53,54,55,56,57] are cited in the supplementary materials.
