# Supplementary material for: Induced Electron Traps via the PCBM in P(VDF-HFP) Composites to Enhance Dielectric and Energy Storage Performance
Source: Polymers (Basel). 2024 Oct 29;16(21):3030. doi: 10.3390/polym16213030 (PMC11548652; doi:10.3390/polym16213030)
Supplement: Supplementary file 1 [file polymers-16-03030-s001.zip › polymers-3267707-supplementary.pdf]

polymers

MDPI

Supporting Materials for the Article

Induced Electron Traps via the PCBM in P(VDF-HFP) Composites to Enhance Dielectric and Energy Storage Performance

Yantao Yang, Jingqi Qiao †, Haiyu Sun ‡, Wenhao Yang, Liangliang Wei and Xuetong Zhao *

State Key Laboratory of Power Transmission Equipment Technology, School of Electrical Engineering, Chongqing University, Chongqing 400044, China; 202311021135t@stu.cqu.edu.cn (Y.Y.); qjq0411@163.com (J.Q.); yuhaisun21shy@163.com (H.S.); 202311131311@stu.cqu.edu.cn (W.Y.); 202311131158t@stu.cqu.edu.cn (L.W.)

* Correspondence: zxt201314@cqu.edu.cn

† Current address: Guangdong Power Grid Corporation, Guangzhou Yuexiu Power Supply Bureau, Guangdong 510030, China.

‡ Current address: Shangqiu Power Supply Company, State Grid Henan Electric Power Company, Henan 476000, China.

S1. Differential scanning calorimetry (DSC) tests

In this work, DSC analysis is used to investigate the crystallization behavior of the P(VDF-HFP)/PCBM composite. Figure S1a presents the melting curves of the P(VDF-HFP)/PCBM composite, where a broad melting peak is observed at around 151°C for both the pristine P(VDF-HFP) and P(VDF-HFP)/PCBM composite. This temperature T_m is defined as the melting temperature. Additionally, a smaller peak around 100 °C is evident, which was commonly attributed to secondary crystallization processes in the amorphous regions [1-3].

Figure S1b shows the crystallization curve based on the cooling process, and the peak refers to the crystallization temperature (T_c). For the pure P(VDF-HFP), T_c is at around 125 °C. It can be seen that T_c first rises and then declines with increasing PCBM content. This increase in T_c is primarily attributed to the introduction of PCBM, which acts as a nucleating agent, promoting heterogeneous nucleation within the PVDF matrix [4].

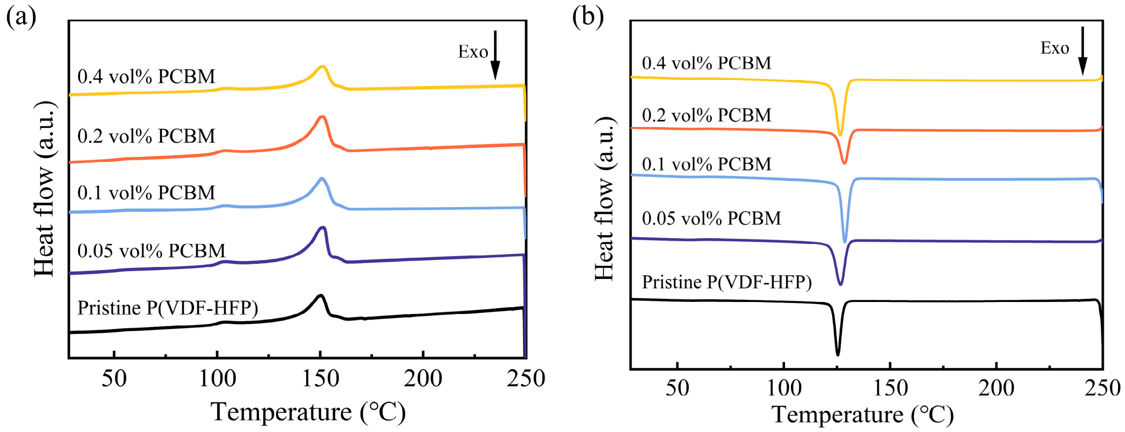

Figure S1. DSC curve of P(VDF-HFP)/PCBM composites, (a) melting curve; (b) crystallization curve.

During the crystallization process, the presence of fillers may impede the motion of polymer chains and influence the polymer's molecular chain mobility, which slows down the stacking and alignment of chains. At low PCBM concentrations, the filler primarily acts as a heterogeneous nucleation agent, promoting nucleation and raising the T_c by accelerating the formation of crystal nuclei. The increase in T_c is due to the additional nucleation sites provided by the PCBM, which facilitate crystallization at higher temperatures. However, as the filler concentration increases beyond the threshold, the movement of polymer chains may become more restricted, leading to slower nucleation and, consequently, a reduction in T_c. Thus, a low concentration of PCBM can raise T_c, while a high concentration may reduce the crystallization temperature because of the physical hindrance of the filler.

The degree of crystallinity (X_c) of the P(VDF-HFP)/PCBM composite can be quantitatively calculated from the DSC data, using the following formula [5]:

$$X_c = \frac{\Delta H_m}{\Delta H_0 (1-\Phi)} \quad (1)$$

where ΔH_m is the measured melting enthalpy (J/g), ΔH_0 is the enthalpy of fusion for fully crystalline P(VDF-HFP) (104.6 J/g), and Φ represents the volume fraction of the filler. The calculated results of T_m, T_c and X_c are shown in Table S1. It is obvious that with the increase of PCBM content, the crystallinity of the composite increases from 19.2 % to the maximum value of 23.4 %. It is indicated that the addition of PCBM induces heterogeneous nucleation and accelerates the nucleation process, resulting in an increase in crystallinity. However, when the PCBM content exceeds 0.4 vol%, X_c decreases, because the excessive PCBM causes non-crystallized regions in the interface areas [6,7].

Table S1. Calculated DSC curves of P(VDF-HFP)/PCBM composites.

Doping concentration (vol%)	T_m (°C)	T_c (°C)	X_c (%)
0	150.3	125.4	19.2%
0.05	151.4	126.8	20.4%
0.1	150.7	128.8	21.1%
0.2	151.2	128.7	23.4%
0.4	151.2	127.1	20.4%

S2. Leakage current properties of the pristine P(VDF-HFP) and P(VDF-HFP)/PCBM composites

Under an applied electric field, leakage current in dielectric materials is resulted from the migration of internal charged carriers. As the electric field increases, more electrons are excited and transported in the composite, leading to an increase in leakage current density. Figure S2a illustrates the relationship between leakage current and the applied electric field for P(VDF-HFP)/PCBM composite films. The results present that the leakage current density of P(VDF-HFP)/PCBM composites is consistently lower than that of pristine P(VDF-HFP) in the applied electrical field from 10-90 MV/m. This reduced leakage current density can be attributed to the trapping effect induced from PCBM, which lowers the internal electric conduction. As a result, the composite exhibits a lower leakage current density compared to the pure P(VDF-HFP).

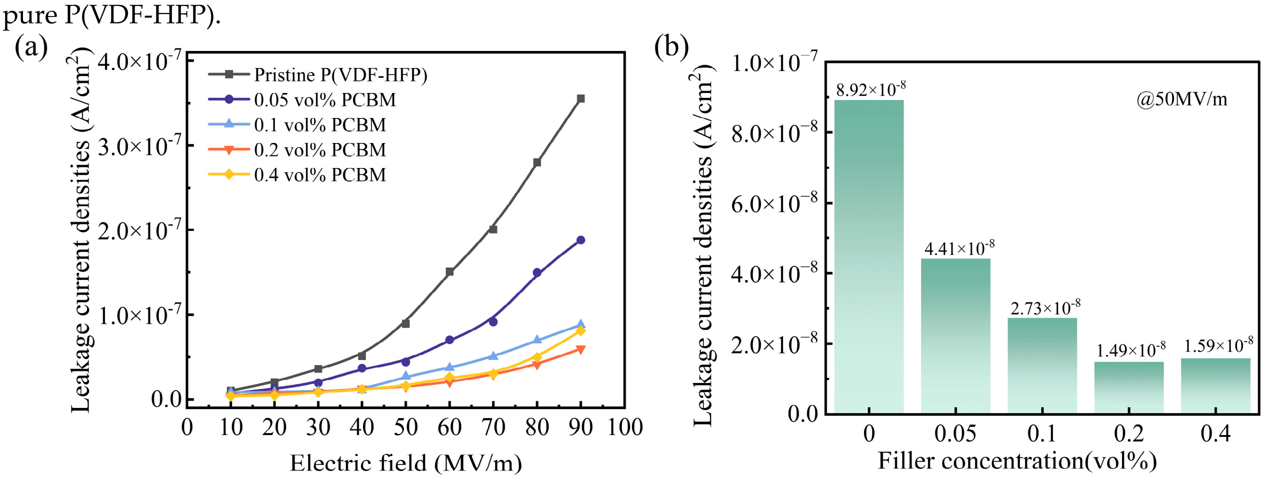

Figure S2. Leakage current density of the pristine P(VDF-HFP) and P(VDF-HFP)/PCBM composites (a) at varied applied electric fields and (b) at an applied electric field of 50 MV/m.

As shown in Figure S2b, under the electric field of 50 MV/m, the leakage current density of the pure P(VDF-HFP) is 8.92×10^{-8} A/cm², whereas the composite containing 0.2 vol% PCBM exhibits a significantly reduced leakage current density of 1.49×10^{-8} A/cm². However, as the PCBM content increases further, the leakage current density begins to rise. When the PCBM doping level reaches 0.4 vol%, the leakage current density increases to 1.59×10^{-8} A/cm². It is suggested that the trapping effect of PCBM becomes weaker at higher doping level, which reduces the charge trapping capability.

References

1. Blundell, D. J. On the interpretation of multiple melting peaks in poly(ether ether ketone). *Polymer* **1987**, *28*, 2248–2251. https://doi.org/10.1016/0032-3861(87)90382-x.
2. Yasuniwa, M.; Tsubakihara, S.; Sugimoto, Y.; Nakafuku, C. Thermal analysis of the double-melting behavior of poly(L-lactic acid). *Journal of Polymer Science Part B Polymer Physics* **2003**, *42*, 25–32. https://doi.org/10.1002/polb.10674.
3. Wu, H.; Qiu, Z. A Comparative Study of Crystallization, Melting Behavior, and Morphology of Biodegradable Poly(ethylene adipate) and Poly(ethylene adipate-co-5 mol % ethylene succinate). *Industrial & Engineering Chemistry Research* **2012**, *51*, 13323–13328. https://doi.org/10.1021/ie301968f.
4. Li, S.; Wang, H.; Liang, X.; Qu, M.; Fan, M.; Zhang, R.; Nakajima, K.; Bin, Y. Structural revolution of PVDF crystallized on MWCNT film on a gradient temperature stage and its dielectric properties. *CrystEngComm* **2024**, *26*, 4458-4469. https://doi.org/10.1039/d4ce00467a.
5. Si, S. K.; Karan, S. K.; Paria, S.; Maitra, A.; Das, A. K.; Bera, R.; Bera, A.; Halder, L.; Khatua, B. B. A strategy to develop an efficient piezoelectric nanogenerator through ZTO assisted γ-phase nucleation of PVDF in ZTO/PVDF nanocomposite for harvesting bio-mechanical energy and energy storage application. *Materials Chemistry and Physics* **2018**, *213*, 525–537. https://doi.org/10.1016/j.matchemphys.2018.04.013.
6. Mendes, S. F.; Costa, C. M.; Sencadas, V.; Nunes, J. S.; Costa, P.; Gregorio, R.; Ribelles, J. L. G.; Lanceros-Méndez, S. Effect of the ceramic grain size and concentration on the dynamical mechanical and dielectric behavior of poly(vinilidene fluoride) $Pb(Zr_{0.53}Ti_{0.47})O_3$ composites. *Applied Physics A* **2009**, *96*, 1037. https://doi.org/10.1007/s00339-009-5323-y.
7. Costa, C. M.; Mendes, S. F.; Sencadas, V.; Ferreira, A.; Gregorio, R.; Ribelles, J. L. G.; Lanceros-Méndez, S. Influence of processing parameters on the polymer phase, microstructure and macroscopic properties of poly(vinilidene fluoride)/$Pb(Zr_{0.53}Ti_{0.47})O_3$ composites. *Journal of Non-Crystalline Solids* **2010**, *356*, 2127–2133. https://doi.org/10.1016/j.jnoncrysol.2010.07.037.
